# Correction: Simple, one-step syntheses of tri- and tetracyclic B,N,S-heterocycles from reactions of a diboracumulene with thiols

**DOI:** 10.1039/d6sc90101h

**Published:** 2026-05-19

**Authors:** Christian Markl, Sourav Kar, Lukas Lubczyk, Kai Hammond, Rian D. Dewhurst, Holger Braunschweig

**Affiliations:** a Institute for Inorganic Chemistry, Julius-Maximilians-Universität Würzburg Am Hubland 97074 Würzburg Germany h.braunschweig@uni-wuerzburg.de; b Institute for Sustainable Chemistry & Catalysis with Boron, Julius-Maximilians-Universität Würzburg Am Hubland 97074 Würzburg Germany

## Abstract

Correction for “Simple, one-step syntheses of tri- and tetracyclic B,N,S-heterocycles from reactions of a diboracumulene with thiols” by Christian Markl *et al.*, *Chem. Sci.*, 2025, **16**, 18911–18918, https://doi.org/10.1039/D5SC04751J.

The authors regret that an incorrect CCDC number (2467670) of compound **3** was included in the manuscript. Also, the crystal data and refinement details for **3** (page 28, SI) were incorrect. The corrected details are shown below, and both the incorrect CIF and SI files have now been updated. These corrections do not affect any of the conclusions of the article.

The corrected CCDC number of compound **3** is 2542695.

Crystal data for **3**. Formula: C_66_H_92_B_2_N_6_S_2_, *M*_r_ = 1055.19, colourless plate, 0.270 × 0.180 × 0.100 mm^3^, monoclinic space group *P*21/*n*, *a* = 10.32070(10) Å, *b* = 22.1980(3) Å, *c* = 26.7343(4) Å, *b* = 99.5610(10)°, *V* = 6039.72(14) Å^3^, *Z* = 4, *r*_calcd_ = 1.160 g cm^−3^, *m* = 1.129 mm^−1^, *F*(000) = 2288, *T* = 100(2) K, *R*_1_ = 0.0765, *wR*_2_ = 0.1702, 11952 independent reflections [2*q* ≤ 151.762°] and 616 parameters.

Refinement details for **3**. The geometry of some phenyl moieties was constrained to the idealized one. The *U*_*ij*_ displacement parameters of atoms C1_4 > C6_5, C1_6 > C6_7, C1_8 > C6_9 were restrained with similarity restraint SIMU.

The Royal Society of Chemistry apologises for these errors and any consequent inconvenience to authors and readers.

